# METTL3-mediated m6A modification increases Hspa1a stability to inhibit osteoblast aging

**DOI:** 10.1038/s41420-024-01925-4

**Published:** 2024-03-27

**Authors:** Yaobin Wang, Yi Chen, Hefang Xiao, Zhongcheng Liu, Xuening Liu, Zhiwei Feng, Xiaoyun Sheng, Bo Peng, Xiaojun Ren, Lihu Xu, Fei Teng, Zhi Yi, YongKang Niu, Dejian Xiang, Yayi Xia, Bin Geng

**Affiliations:** 1 Department of Orthopaedics, Lanzhou University Second Hospital, Lanzhou, Gansu 730030 China; 2 Orthopaedic Clinical Research Center of Gansu Province, Lanzhou, Gansu 730030 China; 3 Intelligent Orthopaedic Industry Technology Center of Gansu Province, Lanzhou, Gansu 730030 China

**Keywords:** Endocrine system and metabolic diseases, Epigenetics, Transcriptomics

## Abstract

Senile osteoporosis is mainly caused by osteoblasts attenuation, which results in reduced bone mass and disrupted bone remodeling. Numerous studies have focused on the regulatory role of m6A modification in osteoporosis; however, most of the studies have investigated the differentiation of bone marrow mesenchymal stem cells (BMSCs), while the direct regulatory mechanism of m6A on osteoblasts remains unknown. This study revealed that the progression of senile osteoporosis is closely related to the downregulation of m6A modification and methyltransferase-like 3 (METTL3). Overexpression of METTL3 inhibits osteoblast aging. Methylated RNA immunoprecipitation sequencing (MeRIP-seq) revealed that METTL3 upregulates the stability of Hspa1a mRNA, thereby inhibiting osteoblast aging. Moreover, the results demonstrated that METTL3 enhances the stability of Hspa1a mRNA via m6A modification to regulate osteoblast aging. Notably, YTH N6-methyladenosine RNA binding protein 2 (YTHDF2) participates in stabilizing Hspa1a mRNA in the METTL3-mediated m6A modification process, rather than the well-known degradation function. Mechanistically, METTL3 increases the stability of Hspa1a mRNA in a YTHDF2-dependent manner to inhibit osteoblast aging. Our results confirmed the significant role of METTL3 in osteoblast aging and suggested that METTL3 could be a potential therapeutic target for senile osteoporosis.

## Introduction

Various factors related to aging can aggravate the development of senile osteoporosis [1, 2]. Cellular aging plays a crucial role in senile osteoporosis as aging cells accumulate in the bone microenvironment over time [3, 4]. Reduced osteogenic differentiation of bone marrow mesenchymal stem cells and impaired function of aging osteoblasts contribute to age-related bone loss [5, 6]. However, these factors alone cannot fully explain the causes of senile osteoporosis. Therefore, the connection between cellular aging and senile osteoporosis should be further investigated to develop effective prevention and treatment approaches.

In recent studies, growing attention has been paid to the role of N6-methyladenosine (m6A) modification in regulating osteoporosis [7–9]. This modification is widely present and conserved in eukaryotic RNA, playing an essential role in various RNA-related biological processes such as transcription, pre-mRNA splicing and processing, nuclear export, translation, RNA stability, and decay [10–12]. Current research on the regulation of osteoporosis by m6A primarily focuses on its effects on bone marrow mesenchymal stem cells (BMSCs). Methyltransferases enhance the osteogenic differentiation of BMSCs by targeting multiple factors [7, 8, 13–15]. Demethylases reduce BMSC osteogenic differentiation via different pathways [16, 17]. Reader proteins increase the stability and protein expression levels of downstream genes related to osteogenesis, promoting the differentiation of BMSCs into osteoblasts [18]. While m6A has been shown to regulate the osteogenic differentiation of BMSCs through various pathways, its direct regulatory role on osteoblasts should not be overlooked. Some studies have reported the regulatory effects of m6A modifications on the differentiation process of osteoblasts [19, 20], but they failed to fully elucidate how m6A directly regulates osteoblast activity or senile osteoporosis.

This study discovered a reduction in m6A modification and methyltransferase-like 3 (METTL3) expression in patients with senile osteoporosis. Further in vitro experiments showed that METTL3 inhibits the aging of osteoblasts, and osteoblast dysfunction affects bone metabolic balance. Subsequently, MeRIP-seq and mRNA-seq were combined, revealing that METTL3 regulates the stability of Hspa1a transcripts. Interestingly, YTH N6-methyladenosine RNA binding protein 2 (YTHDF2) was found to delay the decay of Hspa1a mRNA rather than accelerate its degradation in the process of METTL3 inhibiting osteoblast aging. Moreover, METTL3 specifically overexpressed in mouse osteoblasts and further confirmed that METTL3 can inhibit the progression of senile osteoporosis.

## Results

### Decrease of m6A modification in senile osteoporosis

**Fig. 1:**
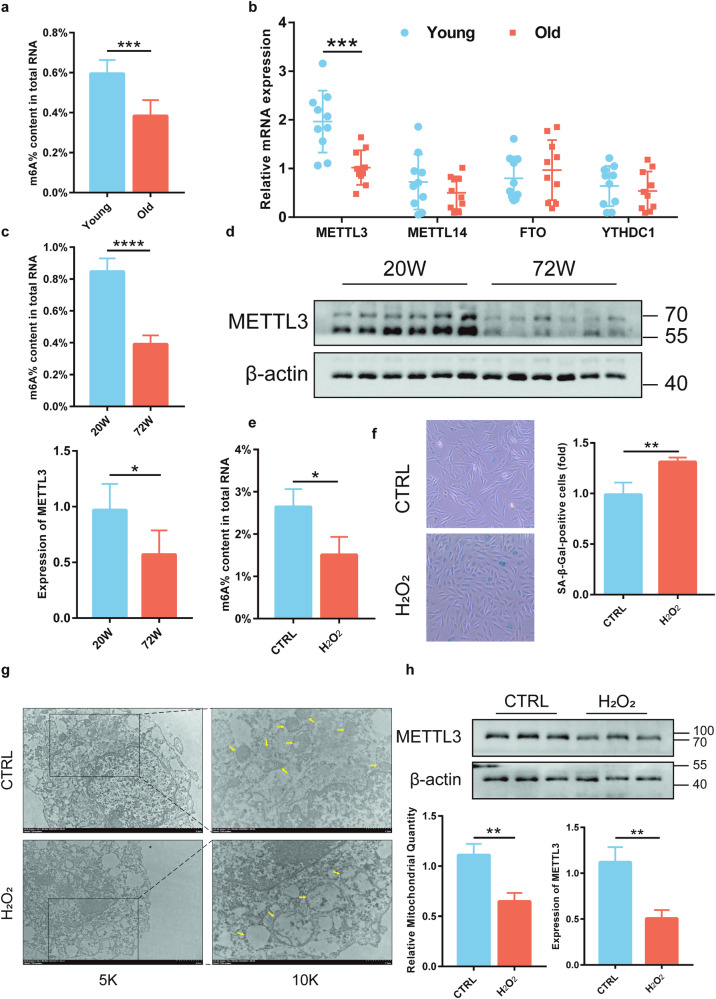
Diminished m6A modification in osteoporosis in the elderly. **a** Colorimetric assessment of m6A levels in bone samples from elderly osteoporosis patients (*n* = 10). **b** qRT-PCR analysis of METTL3, METTL14, FTO, and YTHDC1 expression (*n* = 10). **c** Colorimetric evaluation of m6A levels in mouse osteoporotic bone tissue (*n* = 6). **d** Western blotting to gauge METTL3 protein levels (*n* = 6). **e** Colorimetric analysis of m6A levels in H_2_O_2_-induced aging of MC3T3-E1 cells (n = 3). **f** Identification of senescent cells via β-galactosidase staining (*n* = 3). **g** Transmission electron microscopy for mitochondrial morphology (*n* = 3). **h** METTL3 protein levels were assessed by Western blotting (*n* = 3). Data represented as mean ± standard deviation from three independent experiments. *p < 0.05, **p < 0.01, ***p < 0.001, ****p < 0.0001 by Student’s t-test.

To investigate the relationship between age-related bone loss and m6A modification, colorimetric assays were conducted to measure m6A levels in bone tissues. A significant reduction in m6A modification levels was observed in bone tissues from patients with senile osteoporosis (Fig. 1a). As m6A modification is primarily regulated by methyltransferases and demethylases, the mRNA levels of METTL3, METTL14, FTO, and YTHDC1 were detected in bone tissues. Interestingly, the results revealed a notable decrease in METTL3 mRNA expression specifically in the bone tissues of patients with senile osteoporosis, while no significant change was found in the expression of other genes (Fig. 1b). To further validate our findings, an experimental model of senile osteoporosis was established with 72-week-old mice and the presence of osteoporosis was confirmed using micro-CT scanning and HE staining on the distal femur (Supplementary Fig. 1a, b). Consistently, these aged mice also exhibited significantly reduced levels of m6A modification (Fig. 1c). Additionally, both the mRNA and protein levels of METTL3 were markedly decreased in aged bone tissues (Fig. 1d, Supplementary Fig. 1c).

To investigate the role of METTL3 in aging osteoblasts, an H_2_O_2_-induced MC3T3-E1 model was established (Supplementary Fig. 1d). Our findings revealed several important observations. Firstly, a significant decrease in m6A levels was observed (Fig. 1e), indicating the potential involvement of METTL3 in regulating this modification during osteoblast aging. Furthermore, an increase in SA-β-gal-positive cells was found (Fig. 1f), suggesting cellular senescence. In addition, structural abnormalities were observed within the mitochondria, including swelling, cristae rupture, and vacuolization (Fig. 1g). Lastly, a notable reduction in METTL3 protein expression levels was observed (Fig. 1h). These results suggest that METTL3-mediated m6A modification in osteoblasts may play an essential role in senile osteoporosis.

### METTL3 inhibits in vitro osteoblast aging

**Fig. 2:**
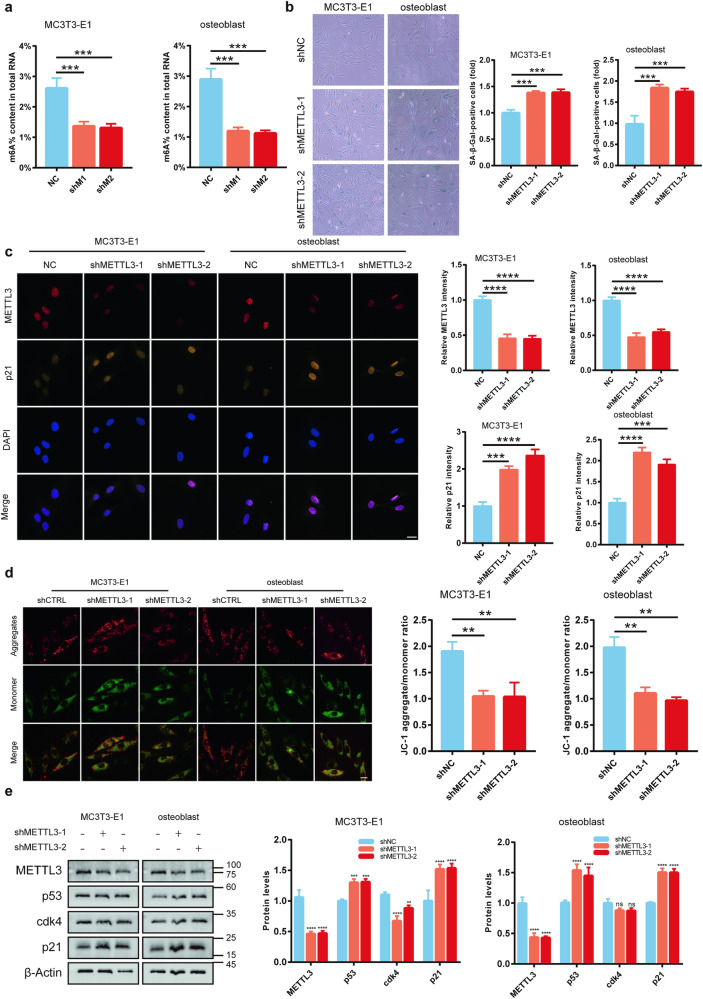
Osteoblast senescence was strongly linked to METTL3-facilitated m6A alterations. **a** Colorimetric assessment of m6A levels in MC3T3-E1 and primary osteoblasts post-METTL3 knockout (*n* = 3). **b** β-Galactosidase staining to identify senescent cells (*n* = 3). **c** Confocal microscopy to reveal METTL3 and p21 localization and expression, bar = 20 μm (*n* = 3). **d** JC-1 staining to measure mitochondrial membrane potential, bar=10 μm (*n* = 3). **e** Western blotting for METTL3, p53, CDK4, and p21 protein levels (*n* = 3). **p < 0.01, ***p < 0.001, ****p < 0.0001, ns, no significant, by Student’s t-test.

Throughout the aging process, various cell types in the bone microenvironment undergo senescence, leading to decreased bone formation and negative bone balance [4, 21]. To investigate whether the senescence of osteoblasts is attributed to reduced m6A modification and decreased METTL3 expression, MC3T3-E1-shMETTL3 was established and METTL3 was knocked down from primary osteoblasts from C57/BL6 mice. We analyzed and identified the differentiation ability of osteoblasts (Supplementary Fig. 1e). The results revealed that m6A levels were significantly downregulated following METTL3 knockout (Fig. 2a), along with a significant increase in SA-β-gal-positive cells (Fig. 2b). Immunofluorescence analysis showed that METTL3 was localized in the cell nucleus, and its silencing led to a significant upregulation of p21 (Fig. 2c). Considering that mitochondrial dysfunction could promote aging [22], the mitochondrial membrane potential in osteoblasts was assessed. After overexpression of METTL3, the ROS of osteoblasts was significantly downregulated (Supplementary Fig. 1F). Compared to the control group, a significant decrease in membrane potential was observed (Fig. 2d), accompanied by upregulated aging-related proteins p53 and p21. In contrast, CDK4 expression was downregulated in MC3T3-E1 cells but showed no significant difference in primary osteoblasts (Fig. 2e). These findings underscore the crucial role of METTL3 in osteoblast aging.

### Effect of osteoblast function on in vitro bone microenvironment metabolism

**Fig. 3:**
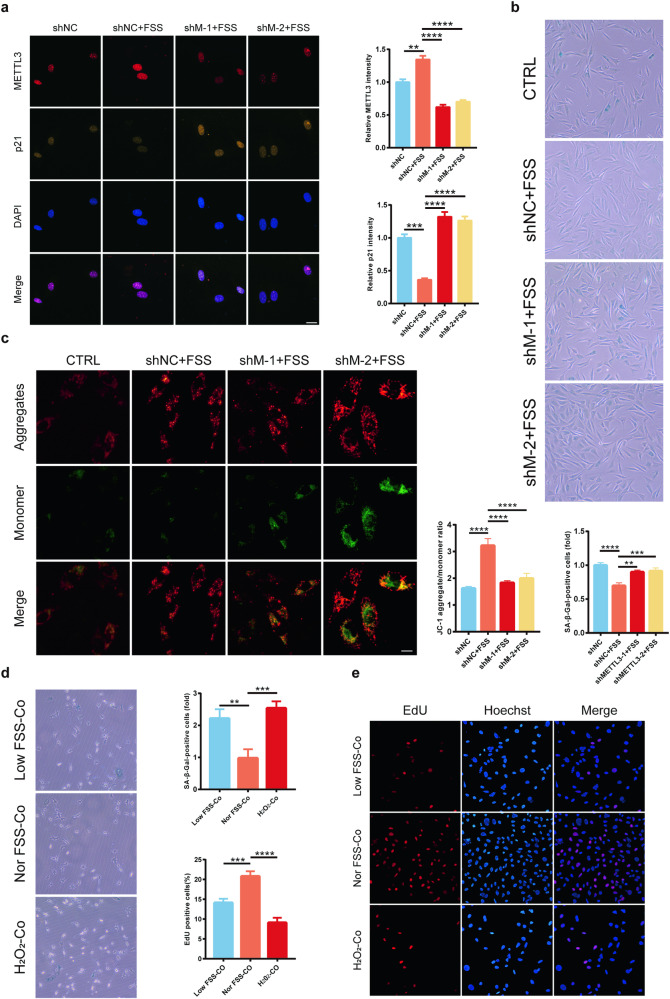
Osteoblast function affects bone microenvironment. **a** Confocal microscopy scans for METTL3 and p21 expression, bar = 20 μm (*n* = 3). **b** β-Galactosidase staining for senescent cells (*n* = 3). **c** JC-1 staining to assess mitochondrial membrane potential, bar = 10 μm (*n* = 3). **d** β-Galactosidase staining for senescent cells (*n* = 3). **e** EdU assay for cellular proliferation, bar = 50 μm (*n* = 3). *p < 0.05, **p < 0.01, ***p < 0.001, ****p < 0.0001 by Student’s t-test.

Senile osteoporosis is a complex condition influenced by various factors that affect the function of osteoblasts and the bone microenvironment [23, 24]. Firstly, the role of METTL3 in osteoblast function was explored by stimulating osteoblast activity. Certain factors, such as hormones [25, 26], growth factors [27, 28], and fluid shear stress (FSS) [29, 30], have been shown to positively stimulate osteoblast activity. Drawing from previous studies [31, 32] and our preliminary findings, FSS has been confirmed to promote the physiological function of osteoblasts, leading to increased bone formation and reduced risk of the developing osteoporosis [33–36]. Therefore, considering all factors, osteoblast function was stimulated through FSS to explore the regulatory role of METTL3. Consistent with our expectations, the immunofluorescence analysis revealed that FSS inhibited various aging phenotypes, including reduced expression of p21 (Fig. 3a), fewer positive SA-β-gal-positive cells (Fig. 3b), and increased mitochondrial membrane potential (Fig. 3c). Interestingly, silencing METTL3 weakened the anti-aging effects of FSS in both cell lines. These results indicated that METTL3 is a key factor affecting the function of osteoblasts.

Subsequently, osteocytes were co-cultured with osteoblasts under low-intensity FSS (60 min 4 dynes/cm^2^), normal-intensity FSS (60 min 12 dynes/cm^2^), or H_2_O_2_-induced osteoblasts to further explore the regulatory role of FSS on the physiological function of bone microenvironment. The osteocytes co-cultured with osteoblasts stimulated by normal-intensity FSS showed an inhibited trend of senescence and higher proliferation activity (Fig. 3d, e). These findings indicated that the physiological functions of osteoblasts are essential for bone formation, and osteoblast aging inhibits bone formation in vitro.

### MeRIP-seq revealed that METTL3 regulates the stability of Hspa1a

**Fig. 4:**
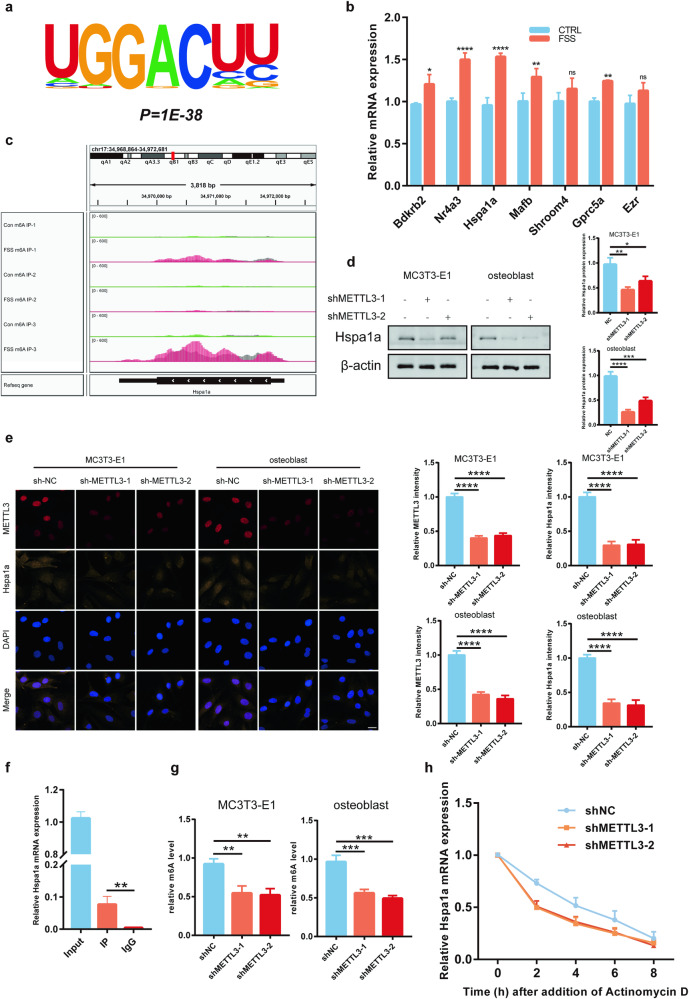
MeRIP-seq reveals that METTL3 regulates the stability of Hspa1a. **a** MeRIP-Seq analysis showed “GGAC” motif enrichment in osteoblasts. **b** qRT-PCR analysis of seven significantly upregulated potential targets (*n* = 3). **c** High m6A peak abundance and specificity near the Hspa1a stop codon. **d** Western blotting to assess Hspa1a protein levels following METTL3 silencing (*n* = 3). **e** Confocal microscopy scans for Hspa1a expression, bar = 20 μm (*n* = 3). **f** RIP-qPCR to investigate direct Hspa1a mRNA-METTL3 protein interactions (*n* = 3). **g** MeRIP-qPCR to explore Hspa1a m6A modifications (*n* = 3). **h** qRT-PCR to measure mRNA degradation after actinomycin D induction (*n* = 3). *p < 0.05, **p < 0.01, ***p < 0.001, ****p < 0.0001 by Student’s t-test.

To explore the potential molecular mechanisms underlying the regulatory role of METTL3 on osteoblast aging, MeRIP-seq and mRNA-seq were performed after activating METTL3 in MC3T3-E1 cells. The “GGAC” sequence was enriched in identified m6A sites in MC3T3-E1 cells (Fig. 4a), with most of the m6A sites located in the CDS and 3’UTR regions (Supplementary Fig. 2a). Metagene analysis showed that m6A sites were mainly located near the stop codons (Supplementary Fig. 2b). A total of 13 significant genes were common in the MeRIP-seq and mRNA-seq analyses (supplementary Fig. 2c). Moreover, the 7 significantly upregulated potential targets were validated, and the qRT-PCR results showed more significant upregulation of Nr4a3 and Hspa1a (Fig. 4b). We found a high abundance and specificity of m6A peaks near the stop codon of Hspa1a (Fig. 4c), suggesting that Hspa1a could be a key regulator in osteoblast aging. Furthermore, SRAMP database [37] prediction was carried out, revealing multiple highly reliable m6A modification sites in Hspa1a mRNA (Supplementary Fig. 2d). In addition, the protein expression levels of Hspa1a were significantly downregulated in both the METTL3-silenced MC3T3-E1 cell line and primary osteoblasts (Fig. 4d). Immunofluorescence also indicated downregulated Hspa1a expression after silencing METTL3 (Fig. 4e). RIP-qPCR results confirmed that Hspa1a mRNA could directly bind with the METTL3 protein (Fig. 4f). Additionally, the MeRIP-qPCR confirmed that the m6A modification levels of Hspa1a mRNA were significantly reduced in both METTL3-silenced cell lines (Fig. 4g). Next, RNA stability experiments were conducted to investigate the mechanism underlying the regulatory effect of METTL3-mediated m6A modification on Hspa1a expression. After METTL3 silencing, the decay of Hspa1a mRNA was significantly faster than the control group (Fig. 4h). These results suggested that METTL3 increases the stability of Hspa1a mRNA and reducing its decay.

### METTL3-mediated m6A modification regulates Hspa1a mRNA decay in a YTHDF2-dependent manner

**Fig. 5:**
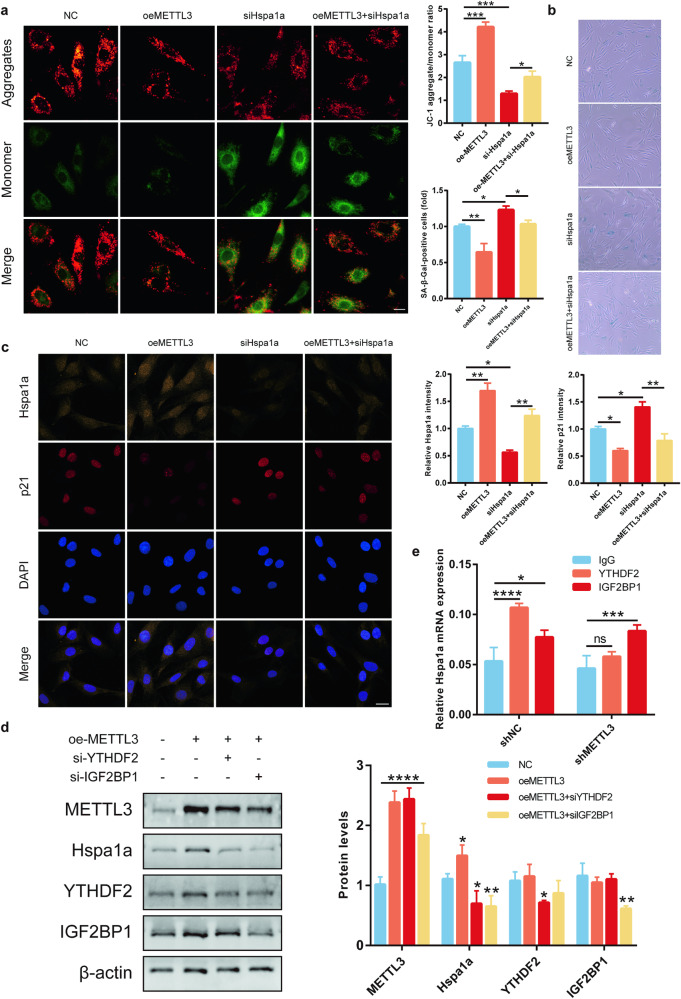
METTL3-driven m6A modification directs YTHDF2-dependent Hspa1a mRNA decay. **a** JC-1 staining to measure mitochondrial membrane potential, bar = 10 μm (*n* = 3). **b** β-Galactosidase staining to identify senescent cells (*n* = 3). **c** Confocal microscopy scans for Hspa1a and p21 expression, bar = 20 μm (*n* = 3). **d** Western blotting for METTL3, Hspa1a, YTHDF2, and IGF2BP1 proteins (*n* = 3). **e** RIP-qPCR to identify m6A-dependent proteins interacting with Hspa1a mRNA (*n* = 3). *p < 0.05, **p < 0.01, ***p < 0.001, ****p < 0.0001, ns, no significant, by Student’s t-test.

After overexpressing METTL3, we knocked down Hspa1a and validated the knockdown efficiency (Supplementary Fig. 2e). Knockdown of Hspa1a suppressed the regulatory effects of METTL3 on osteoblast aging, including the downregulation of mitochondrial membrane potential (Fig. 5a), increased β-galactosidase stained-positive cells (Fig. 5b), and upregulated expression of the aging-associated molecule p21 (Fig. 5c). These findings indicated that Hspa1a is a downstream target of methyltransferase METTL3 and is essential in modulating osteoblast aging through m6A modification.

The functional role of m6A-modified RNA is primarily mediated by “readers,” which mainly includes YTH domain family proteins (YTHDF1, YTHDF2, YTHDF3, YTHDC1, YTHDC2) and the insulin-like growth factor 2 mRNA-Binding protein (IGF2BP) family (IGF2BP1, IGF2BP2, IGF2BP3) [38, 39]. Considering that METTL3 primarily regulates the stability of Hspa1a mRNA, YTHDF2 and IGF2BP1 for validation. Interestingly, the knockdown of both YTHDF2 and IGF2BP1 were selected significantly inhibited the METTL3-induced increase in Hspa1a protein expression (Fig. 5d). Further RIP-qPCR analyses revealed that Hspa1a directly interacts with YTHDF2 but not with IGF2BP1 (Fig. 5e). These findings suggest that the m6A-modified mRNA of Hspa1a by METTL3 is a target of YTHDF2. In summary, our results indicate that METTL3 regulates Hspa1a expression in a YTHDF2-dependent manner.

### Targeting METTL3 in osteoblasts inhibits the progression of senile osteoporosis

**Fig. 6:**
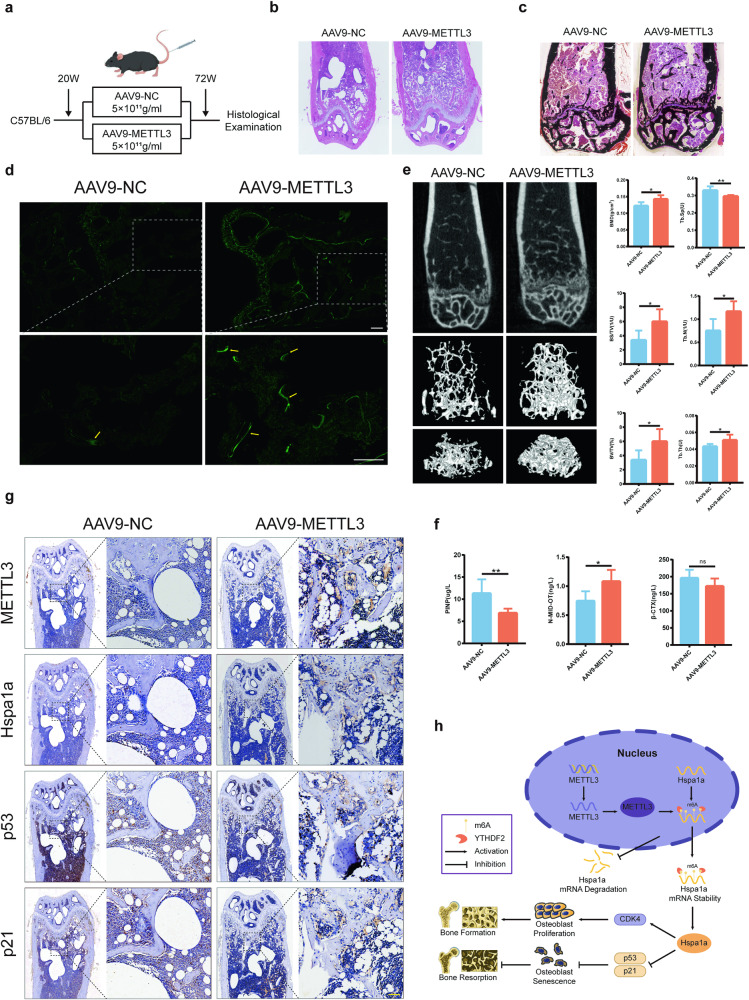
METTL3 targeting in osteoblasts inhibits osteoporosis in aged mice. **a** Diagrammatic representation of mouse groups and interventions (*n* = 6). **b**, **c**, **f** Histological evaluations (HE staining, Von Kossa staining, and calcein staining, bar = 200 μm) of the distal femur (*n* = 3). **d** Quantitative micro-CT analysis of the distal femur (*n* = 3). **e** ELISA for serum bone metabolic markers (*n* = 3). **g** Immunohistochemistry for METTL3, Hspa1a, p53, and p21 levels in bone tissue (*n* = 3). **h** Schematic diagram showing METTL3-mediated m6A modification regulates Hspa1a mRNA decay in a YTHDF2-dependent manner, and METTL3 controls the progression of osteoporosis in the elderly by inhibiting bone absorption through osteoblast senescence. bar = 50 μm. *p < 0.05, **p < 0.01, ns, no significant, by Student’s t-test.

To validate whether targeting METTL3 in osteoblasts could inhibit the progression of senile osteoporosis, osteoblast-specific AAV9-METTL3 was administered via tail vein injection to 20-week-old C57BL/6 J mice (Fig. 6a, Supplementary Fig. 2f). HE staining revealed a significant decrease in trabecular number and exhibited fractures of the trabeculae, failing to form a network, and having extensive lipid droplet deposition in the NC group compared to the AAV9-METTL3 group (Fig. 6b). In addition, Von Kossa staining showed increased bone mineral deposition in the AAV9-METTL3 group (Fig. 6c), suggesting enhanced osteogenic potential. Calcein staining indicated increased new bone formation in the AAV9-METTL3 group (Fig. 6d). Micro-CT analysis revealed that after AAV9-METTL3 injection, the parameters BMD, BV/TV, BS/TV, Tb.TH, and Tb.N significantly increased, while Tb.Sp demonstrated a significant decrease (Fig. 6e), thereby delaying the progression of senile osteoporosis. Serum ELISA results showed a significant upregulation in N-MID-OT and a significant downregulation in PINP, while the downregulation of β-CTx was not statistically significant (Fig. 6f), suggesting that METTL3 primarily affects bone-forming ability in senile osteoporosis. Furthermore, the IHC results showed that after overexpressing METTL3 specifically targeting osteoblasts, Hspa1a expression was significantly upregulated, while p53 and p21 were significantly downregulated at the distal femoral trabecular region (Fig. 6g). These results indicated that specific targeting of METTL3 in osteoblasts could inhibit senile osteoporosis.

## Discussion

Normal bone metabolism is determined by the dynamic balance between bone formation and resorption [40, 41]. With age, osteoblasts exhibit a functional decline and reduced numbers, leading to decreased bone formation, compromised structural integrity, and elevated risks of osteoporosis and fractures [42, 43]. Therefore, osteoblast aging remains a critical factor in senile osteoporosis. The current study is the first to report the regulatory mechanism of m6A modifications on osteoblast aging and senile osteoporosis. Our research makes three notable contributions. First, m6A modifications were found to be downregulated in bone tissues and osteoblasts in senile osteoporosis, and METTL3 was identified as the key factor behind this downregulation. Second, METTL3 was found to play a vital role in osteoblast aging by modulating the expression of Hspa1a through m6A modifications. Finally, the METTL3-mediated decay of Hspa1a mRNA was found to be regulated in a YTHDF2-dependent manner. These findings suggested that the molecular pathway involved in METTL3-mediated m6A modifications in osteoblasts may serve as a potential therapeutic target for senile osteoporosis.

m6A modification is an epigenetic modification that plays a crucial role in bone development and metabolism [9, 44]. Recent studies have shown that m6A regulates various bone cells, thereby influencing the development of osteoporosis39, 40. Moreover, METTL3 regulates osteoclast differentiation and function by controlling RNA stability and nuclear export [44, 45]. METTL3 has also been found to modulate the progression of BMSCs and osteoporosis. Conditional knockout of METTL3 in BMSCs has been shown to increase bone loss, leading to impaired bone formation and osteoporotic pathological features [7]. Additionally, the lack of METTL3 has been reported to inhibit the osteogenic differentiation potential of BMSCs [14]. This study focused on m6A modifications in senile osteoporosis and in osteoblasts. Our research showed significantly reduced m6A modification levels and markedly downregulated METTL3 expression levels in senile osteoporosis patients. The senile osteoporosis mouse model was established, revealing that the m6A modification levels in the bone tissues of these mice were also significantly reduced. These findings were consistent with the significant reduction in both the mRNA and protein levels of METTL3 in aging bone tissues. Therefore, our study supports the regulatory role of m6A and METTL3 in senile osteoporosis.

To further explore the regulatory relationship between METTL3-mediated m6A methylation and senile osteoporosis, METTL3 was silenced and then restored in vitro to validate its effects on osteoblast function. Inhibition of METTL3 accelerated osteoblast aging, while FSS stimulation partially delayed aging. Notably, when aging osteoblasts were co-cultured with osteocytes in vitro, aging osteoblasts were found to inhibit the physiological activity of osteocytes. These results confirmed that aging osteoblasts impact the balance of bone metabolism in the bone microenvironment.

Furthermore, MeRIP-seq and mRNA-seq screening were performed to identify the target by which METTL3-mediated m6A modification inhibits osteoblast aging; the results revealed that Hspa1a was a molecular target of METTL3. Hspa1a, also known as heat shock protein 70 (HSP70), is involved in various cellular processes including protein folding, stress responses, and cellular injury protection [46–48]. One study has shown that Hspa1a is related to DNA repair and protects osteoblasts from genotoxic stress [49]. Another study demonstrated increased Hspa1a expression during osteogenic differentiation of rat bone marrow stromal cells, which mediated the expression of bone-specific proteins such as ALP, RUNX2, OCN, and COL1A1 [50]. These studies suggested that Hspa1a plays a protective and bone-promoting role in osteoporosis, but whether Hspa1a is involved in regulating osteoblast aging remained unelucidated. Firstly, Hspa1a was confirmed to be a direct molecular target of METTL3-mediated m6A modification in regulating osteoblast aging through MeRIP and RIP. Subsequently, Hspa1a was inhibited and overexpressed in vitro, confirming that Hspa1a can inhibit osteoblast aging. These results indicated that METTL3-mediated m6A modification increases the stability of Hspa1a mRNA, thereby inhibiting osteoblast aging. Interestingly, a recent study has shown that the m6A demethylase FTO directly targets Hspa1a and promotes osteogenic differentiation, maintaining bone mass. This supports our research findings on Hspa1a inhibiting osteoblast aging and further elucidates the dynamic diversity of m6A modification in regulating osteoporosis. However, methyltransferases and demethylases are not unchangeable and absolutely opposite. Osteoblasts express METTL3 to counteract osteoblast aging, thereby inhibiting the reduction of bone formation; expressing FTO protects osteoblasts from genetically induced apoptosis, protecting bone mass. Therefore, this subject warrants further in-depth exploration.

The role of reading proteins in m6A modification is a key link that cannot be ignored. Subsequently, YTHDF2 and IGF2BP1 were verified to regulate the stability of mRNA. Surprisingly, both of them resulted in increased Hspa1a protein levels. As opposed to IGF2BP1, the well-known YTHDF2 often suppresses the stability of transcripts and aggravates its decay [51–53]. However, some new studies have demonstrated that YTHDF2 stabilizes the targeted transcript in an m6A-dependent manner [54, 55]. Furthermore, YTHDF2 suppressed the attenuation of Hspa1a mRNA by RIP-PCR directly at the mRNA level. Mouse osteoblasts were specifically targeted for METTL3 overexpression, which effectively delayed the age-related reduction of bone formation and inhibited the progression of senile osteoporosis.

In summary, our study provides new evidence of the essential role of METTL3 in inhibiting osteoblast senescence and the progression of senile osteoporosis. METTL3 inhibits osteoblast senescence by increasing the stability of Hspa1a mRNA through YTHDF2-dependent m6A modification (Fig. 6h). In addition, this study emphasizes the importance of m6A modification in osteoblasts in senile osteoporosis, which provides new insights into the potential molecular mechanism of METTL3 regulating osteoblast senescence and the development of strategies for the treatment of senile osteoporosis.

## Methods

### Patient sample collection

This study was approved by the Research Ethics Committee of Lanzhou University Second Hospital (Approval No. 2022A-714) and conducted in accordance with the Declaration of Helsinki. Trabecular bone samples of senile osteoporosis were obtained from 10 patients who underwent total hip replacement surgery for osteoporotic femoral neck fractures at Lanzhou University Second Hospital. Control trabecular bone samples were obtained from 10 patients, undergoing arthroscopic anterior cruciate ligament repair, with no osteoporosis. Informed consent was obtained from all subjects. Detailed patient information is provided in Supplementary Tables S1, 2.

### Mice

All female C57BL/6 J mice (20 weeks, 72 weeks) were purchased from the Lanzhou Veterinary Research Institute, Chinese Academy of Agricultural Sciences, and raised under specific pathogen-free conditions. Animal sample size assessment is based on the law of diminishing returns. Bone tissues were collected from euthanized 72-week-old and 20-week-old mice for further study (*n* = 6). For in vivo experiments, 20-week-old C57BL/6 J mice were randomly divided into AAV9-METTL3 group (*n* = 6) or AAV9-NC group (*n* = 6) using random number table method. Mice were euthanized 52 weeks post-injection, and serum and bone tissue samples were collected. Calcein staining was performed 10 and 3 days before euthanasia by injecting 10 ug/g of calcein. Mice and samples were digitally labeled to ensure operator blinded. All animal experiments were approved by the Animal Experiment Management Committee of Lanzhou University Second Hospital (Approval No. D2022-078).

### Cell culture and co-culture

The mouse cell line MC3T3-E1 was purchased from the National Infrastructure of Cell Line Resource and cultured in α-MEM medium (Gibco) supplemented with 10% fetal bovine serum (FBS, Gibco) at 37 °C and 5% CO_2_. The mouse cell line MLO-Y4 was purchased from Fuheng Biology and was cultured under the same conditions as the MC3T3-E1 cell line. Primary osteoblasts were isolated from the calvaria of C57BL/6 J neonatal mice using the tissue explant method and cultured in DMEM (Gibco) supplemented with 10% FBS at 37 °C and 5% CO_2_. Osteoblast aging models were established by treating cells with 200 μM H_2_O_2_ for 4 h. For co-culture, osteocytes were seeded in 6-well plates, and a 24 mm diameter tissue culture insert with a 0.4 μm pore size was placed on top, containing osteoblasts subjected to low fluid shear stress, normal fluid shear stress, and H_2_O_2_-induced osteoblasts.

### Plasmid and siRNA transfection

Knockout and overexpression of METTL3 and Hspa1a were carried out using lentivirus constructs following the manufacturer’s instructions (Genechem). For in vivo overexpression of METTL3, osteoblast-specific AAV9-METTL3 was injected via the tail vein following the manufacturer’s instructions (Genechem). In addition, siRNAs for YTHDF2 and IGF2BP1 were transfected according to the manufacturer’s protocol (GenePharma).

### Measurement of m6A RNA methylation

Total RNA was extracted from clinical samples, mouse samples, and cell samples. 100–300 ng of RNA was used as input for the m6A RNA methylation level assay according to the manufacturer’s instructions (AmyJet Scientific).

### qRT-PCR

Total RNA was extracted from cells and tissues using Trizol (Accurate Biology). cDNA was prepared using an Evo M-MLV RT PreMix kit with gDNA removal (Accurate Biology). qRT-PCR was performed on an LC480 system (Roche) using SYBR Green Pro Taq HS (Accurate Biology). GAPDH was used as an internal control. Specific primers used for target gene expression are listed in Supplementary Table S3.

### Western blot analysis

Total protein was extracted from cells or tissues using RIPA lysis buffer with protease inhibitor cocktail (Beyotime Biotech). Protein concentration was measured using a BCA Protein Assay Kit (Beyotime Biotech) and analyzed by SDS-PAGE. Antibodies used in this study were METTL3 (abcam, ab195352, 1:1000), p53 (CST, 2524 T, 1:1000), Cdk4 (abcam, ab199728, 1:2000), p21 (abcam, ab109199, 1:1000), Hspa1a (abcam, ab5439, 1:1000), β-actin (abcam, ab8226, 1:5000), YTHDF2 (Proteintech, 24744-1-AP), and IGF2BP1 (Proteintech, 22803-1-AP). Uncropped gel images are provided in the supplementary material.

### MeRIP-seq and RNA-seq

Total RNA was extracted from MC3T3-E1 cells subjected to 60 min of 12 dynes/cm^2^ fluid shear stress using Trizol (Invitrogen). After RNA fragmentation, m6A antibody (Millipore) enrichment was performed, followed by purification. Library construction for IP and Input samples was performed using the SMARTer® Stranded Total RNA-Seq Kit v2- Pico Input Mammalian User Manual (Takara), and sequencing was done on the Illumina platform.

### RNA stability assay

To explore the stability of Hspa1a mRNA in MC3T3-E1 NC and shMETTL3 cells, actinomycin D (Selleck) was added to the complete medium at 5 μg/ml. Cells were collected at specified times, and RNA was extracted for qRT-PCR to determine the half-life of Hspa1a.

### m6A RIP-qPCR and RIP-qPCR analysis

The level of m6A modification on Hspa1a mRNA was detected using a MeRIPTM m6A Transcriptome Profiling Kit (RiboBio), following the same method as MeRIP-seq. Primers for qRT-PCR are listed in Supplementary Table S4. RIP analysis was performed using a Magna RIP™ RNA-Binding Protein Immunoprecipitation Kit. Briefly, the cells were treated with RIP lysate containing protease inhibitor cocktail and RNase inhibitor. Then the magnetic beads coupled with METTL3, YTHDF2, igG, or IGF2BP1 antibodies were collected and incubated overnight at 4 °C. RNA-protein complex was extracted by magnetic beads and protein was digested by protease K. Finally, RNA was purified and qRT-PCR was detected.

### SA-β-Gal staining

Cellular senescence was detected using a Senescence β-Galactosidase Staining Kit (Beyotime Biotech). After washing with PBS, cells were fixed for 15 min at room temperature and then incubated overnight at 37 °C with SA-β-Gal reaction solution. Imaging was performed using EVOS XL Core (Thermo Fisher Scientific, America).

### JC-1 staining

Cells were incubated with 1 ml of JC-1 staining working solution in 1 ml of complete culture medium at 37 °C for 20 min. After washing with JC-1 staining buffer twice, 2 ml of cell culture medium was added. Imaging was performed using an OLYMPUS microscope (Japan).

### EdU

EDU staining is carried out according to the instructions of the manufacturer (RiboBio). To put it simply, the treated cells were first incubated in a medium containing 50 μ MEDU for 3 h. Then it was fixed and permeated, and finally stained and incubated. OLYMPUS microscope (Japan) was used for shooting and imaging.

### Immunofluorescence

Cells were washed with PBS, fixed with 4% paraformaldehyde at room temperature for 30 min, and permeabilized with 0.1% Triton X-100 at room temperature for 30 min. After blocking with 10% goat serum at 37 °C for 1 h, cells were incubated with primary antibodies overnight at 4 °C. After washing, cells were incubated with fluorescent secondary antibodies at 37 °C for 1 h. Nuclei were stained with DAPI. Imaging was performed using confocal laser scanning microscopy (Carl Zeiss).

### Histology

Mouse femoral distal ends were fixed in 4% paraformaldehyde for 3 days for Micro-CT analysis. Decalcification was performed using EDTA decalcifying solution (Solarbio) for 30 days, followed by ethanol gradient dehydration and paraffin embedding. 5 μm sections were prepared for HE staining and IHC.

### Micro-quantitative computed tomography analysis (Micro CT)

Mouse femur samples were fixed in 70% ethanol and scanned using a micro-CT (Scanco Viva CT 8.0). Three-dimensional reconstruction images were generated, and bone structural parameters including Bone Mineral Density (BMD), Trabecular Number (Tb.N), Trabecular Thickness (Tb.Th), Trabecular Separation (Tb.Sp), Bone Volume/Tissue Volume (BV/TV), and Bone Surface/Bone Volume (BS/BV) were analyzed.

### ELISA

Mouse serum samples stored at −80 °C were slowly thawed on ice. After preparing a standard curve, standard and test samples were added and incubated at 37 °C for 30 min. After washing, enzyme-labeled reagents were added and incubated at 37 °C for 30 min. After another wash, chromogenic reagents were added and incubated at 37 °C for 10 min in the dark. Finally, the stop solution was added, and absorbance at 450 nm was measured.

### Statistical analyses

Results were shown as mean ± SD from at least three independent experiments. Data were subjected to statistical analyses using SPSS 22.0 software (IBM Corp.) The results were expressed as mean ± SD. The Student’s t-test was used for comparisons between two groups, and single factor analysis of variance was used for multi-group comparisons. In this study, *P* < 0.05 was considered statistically significant.

### Supplementary information

Supplementary Figures

Supplementary Information Legends

SUPPLEMENTAL MATERIAL

## Data Availability

All data are available from the corresponding author upon reasonable request.
