# Supplementary figures and images for: METTL3-mediated m6A modification increases Hspa1a stability to inhibit osteoblast aging
Source: Cell Death Discov. 2024 Mar 27;10:155. doi: 10.1038/s41420-024-01925-4 (PMC10973419; doi:10.1038/s41420-024-01925-4)

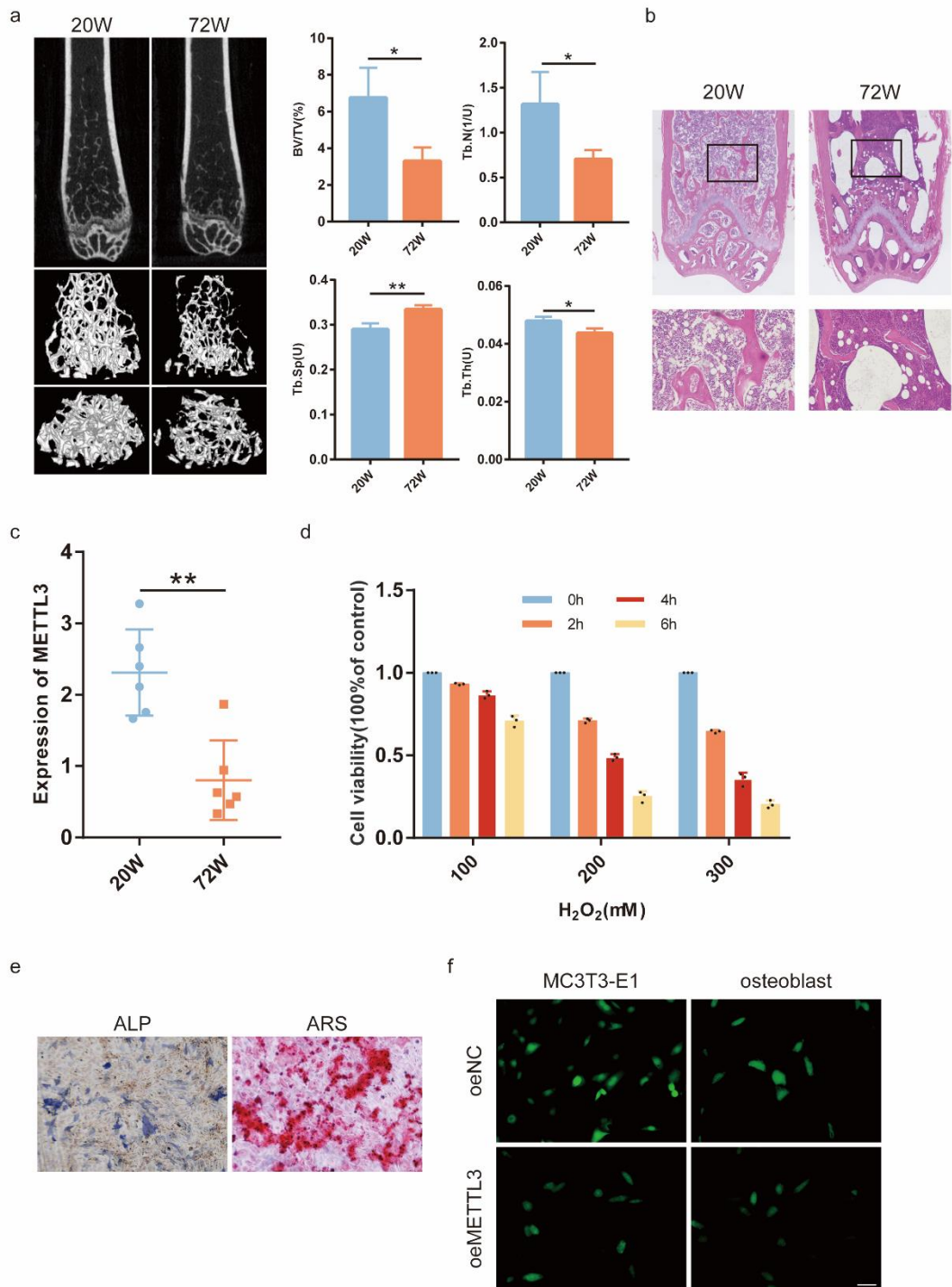

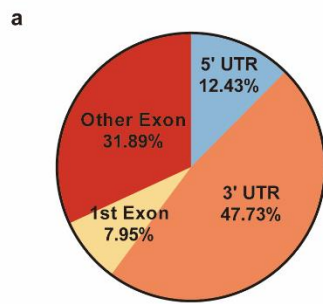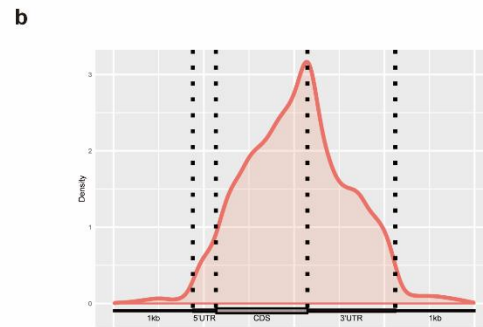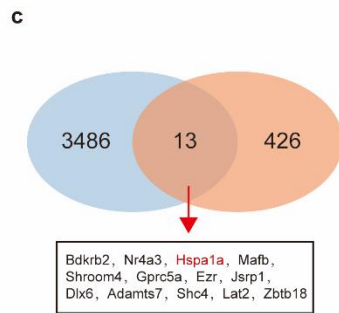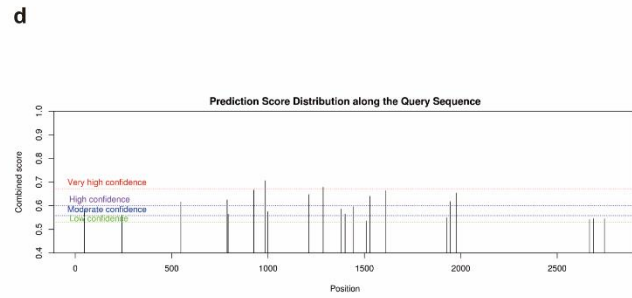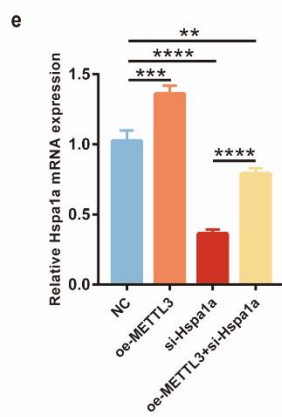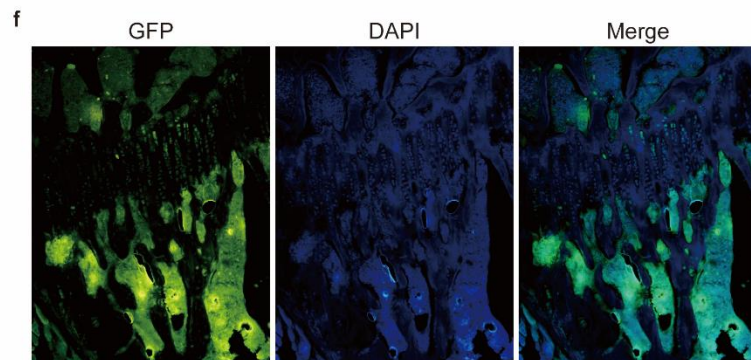

Supplement: Supplementary file 1 — Supplementary Figures [file 41420_2024_1925_MOESM1_ESM.pdf]
