# Supplementary material for: METTL3-mediated m6A modification increases Hspa1a stability to inhibit osteoblast aging
Source: Cell Death Discov. 2024 Mar 27;10:155. doi: 10.1038/s41420-024-01925-4 (PMC10973419; doi:10.1038/s41420-024-01925-4)
Supplement: Supplementary file 2 — Supplementary Information Legends [file 41420_2024_1925_MOESM2_ESM.docx]

**Supplementary Information Legends**

**Supplementary Figure 1:**(a) μCT images of the distal femur (left), quantitative μCT analysis of distal femur trabecular bone (n=3). (b) HE staining of the distal femur (n=3). (c) METTL3 mRNA expression levels measured by qRT-PCR (n=10). (d) Cell viability measured by CCK-8 (n=3). (e) Identification of differentiation ability of primary osteoblasts. (f) oe-METTL3 downregulated ROS levels. bar=20 μm (n=3) **p*<0.05, ***p*<0.01 by Student’s t-test.

**Supplementary Figure 2:**(a) Major m6A sites in osteoblasts are located in the CDS and 3'UTR regions. (b) Metagene analysis showing m6A sites mainly located near the stop codon. (c) 13 significantly changed genes enriched after MeRIP-seq and mRNA-seq analysis. (d) m6A modification sites on Hspa1a mRNA predicted by SRAMP database. (e) qRT-PCR analysis of Hspa1a expression (n=3). (f) AAV9-METTL3 targeting osteoblasts. ****p*<0.001, *****p*<0.0001 by Student’s t-test.

**Supplementary Figure 3:** Supplementary Information-Uncropped Immunoblots.

**Supplementary Table S1.** Information of the patients with senile osteoporosis.

**Supplementary Table S2.** Information of the patients with non-senile osteoporosis.

**Supplementary Table S3.** Primers used for qPCR, primer sequence (5'-3').

**Supplementary Table S4.** Primers used for m6A MeRIP-qPCR analysis, primer sequence (5'-3').
