# Supplementary material for: METTL3-mediated m6A modification increases Hspa1a stability to inhibit osteoblast aging
Source: Cell Death Discov. 2024 Mar 27;10:155. doi: 10.1038/s41420-024-01925-4 (PMC10973419; doi:10.1038/s41420-024-01925-4)
Supplement: Supplementary file 3 — SUPPLEMENTAL MATERIAL [file 41420_2024_1925_MOESM3_ESM.doc]

Table S1. Information of the patients with senile osteoporosis

| Patient Number | Gender | Age | BMD (T-score) |
| --- | --- | --- | --- |
| 1 | Male | 76 | -2.84 |
| 2 | Female | 79 | -3.32 |
| 3 | Female | 75 | -2.81 |
| 4 | Male | 83 | -4.10 |
| 5 | Female | 71 | -2.69 |
| 6 | Female | 76 | -3.48 |
| 7 | Male | 74 | -3.64 |
| 8 | Female | 75 | -2.95 |
| 9 | Male | 75 | -2.68 |
| 10 | Female | 77 | -3.56 |

Table S2. Information of the patients with non-senile osteoporosis

| Patient Number | Gender | Age | BMD (T-score) |
| --- | --- | --- | --- |
| 1 | Male | 37 | -1.50 |
| 2 | Male | 46 | -1.10 |
| 3 | Female | 41 | -2.13 |
| 4 | Male | 35 | 0.65 |
| 5 | Female | 36 | -1.44 |
| 6 | Female | 38 | -1.36 |
| 7 | Male | 32 | 0.92 |
| 8 | Male | 41 | -1.06 |
| 9 | Female | 43 | -1.42 |
| 10 | Female | 35 | -1.39 |

| Table S3. Primers used for qPCR | | |  |
| --- | --- | --- | --- |
|  | Primer sequence(5'-3') | |  |
| Target gene | Forward | Reverse |  |
|  |
| Mouse Bdkrb2 | CATCGCCAATAACTTTGACTGG | CTGCTGTACAGGTTCATGTAGA |  |
| Mouse Nr4a3 | TTGATCAAGATGGAAGAGGGTC | GAGACTGCTTGAAGTACATGGA |  |
| Mouse Mafb | GAAACATCACCTGGAGAACGAG | TTTCTCGCACTTGACCTTGTAG |  |
| Mouse Shroom4 | CAGCAGCAGCAGCAACAACAAC | GGTCCTCCTCTTTCTCTCCTTCCTC |  |
| Mouse Hspa1a | GGTGCTGACGAAGATGAAGGAGATC | CTGCCGCTGAGAGTCGTTGAAG |  |
| Mouse Gprc5a | CTGACCTTCTTCGCATCCTTCTTGG | GCAATGGAGAGGAACGAGGTGAAG |  |
| Mouse Ezr | AAGTTTGTCATTAAGCCCATCG | AGGATCCGCTTGTTAATTCTCA |  |
| Human METTL3 | CTTCAGCAGTTCCTGAATTAGC | ATGTTAAGGCCAGATCAGAGAG |  |
| Human FTO | GTTCACAACCTCGGTTTAGTTC | CATCATCATTGTCCACATCGTC |  |
| Human YTHDC1 | AGTGACTCTGGTTCTGAATCTG | CTGGTTTGATCTTTTCGGACAG |  |
| Human METTL14 | ACCAAAATCGCCTCCTCCCAAATC | AGCCACCTCTTTCTCCTCGGAAG |  |

| Table S4. Primers used for m6A MeRIP-qPCR analysis | | |  |
| --- | --- | --- | --- |
|  | Primer sequence(5'-3') | |  |
| Gene | Forward | Reverse |  |
|  |
| Hspa1a | GGTGCTGACGAAGATGAAGGAGATC | CTGCCGCTGAGAGTCGTTGAAG |  |
